# Limbal stem cell deficiency: a dreaded complication of chickenpox in children

**DOI:** 10.1093/omcr/omaf037

**Published:** 2025-05-28

**Authors:** Oumayma Elmansouri, Assia Lemkhoudem, Houda Bezza, Houssaine Ait Lhaj, Mohamed Kriet, Fouad Elasri

**Affiliations:** Department of ophthalmology, Avicenna Military hospital, Ain Mezouar, Gueliz, Marrakesh 40000, Morocco; Department of ophthalmology, Avicenna Military hospital, Ain Mezouar, Gueliz, Marrakesh 40000, Morocco; Department of ophthalmology, Avicenna Military hospital, Ain Mezouar, Gueliz, Marrakesh 40000, Morocco; Department of ophthalmology, Avicenna Military hospital, Ain Mezouar, Gueliz, Marrakesh 40000, Morocco; Department of ophthalmology, Avicenna Military hospital, Ain Mezouar, Gueliz, Marrakesh 40000, Morocco; Department of ophthalmology, Avicenna Military hospital, Ain Mezouar, Gueliz, Marrakesh 40000, Morocco

**Keywords:** paediatrics, infectious diseases and tropical medicine, medical ophthalmology

## Abstract

We report a rare case of Limbal Stem Cell Deficiency (LSCD) in a 10-year-old child following chickenpox. The child presented with persistent ocular symptoms, including pain, redness, and photophobia, two months after the resolution of systemic chickenpox. Ophthalmic examination revealed conjunctival hyperemia, 360° conjunctivalization, corneal neovascularization, and an epithelial defect. LSCD, characterized by corneal neovascularization and conjunctivalization, can occur after viral infections, including herpes zoster. To our knowledge, this is the first reported case of LSCD following chickenpox in a child. Early recognition and collaborative care between pediatricians and ophthalmologists are essential for effective management.

We report the case of a 10-year-old child presenting with a painful red left eye with lacrimation and photophobia evolving for two months. These ocular symptoms coincided with the onset of a generalized rash and fever, leading to a clinically confirmed diagnosis of chickenpox by the pediatrician. The patient was started on oral aciclovir and ocular lubricants and vitamin A ointment. However, the ocular symptoms persisted even after the resolution of systemic chickenpox symptoms.

Ophthalmological examination of the left eye revealed an AV of 9/10, conjunctival hyperemia, 360° conjunctivalization and neovascularization of the cornea, sparing the visual axis. The fluorescein test was positive, revealing an epithelial defect (Fig. 1). The rest of the anterior segment and fundus examination was unremarkable.

**Figure 1:**
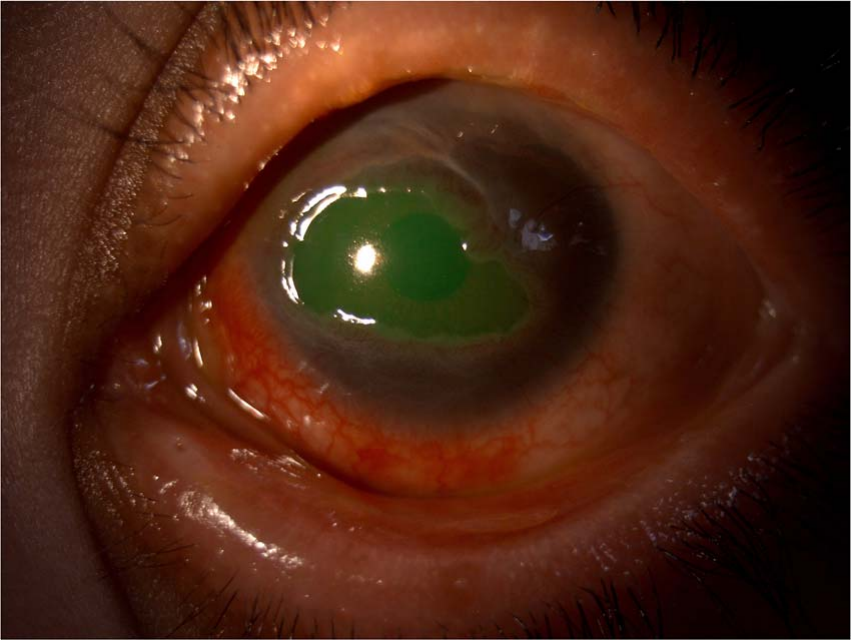
Conjunctival hyperemia, 360° conjunctivalization and neovascularization of the cornea, sparing the visual axis. The fluorescein staining reveals an epithelial defect in a 10-year-child with a history of chickenpox.

The treatment approach includes lubricants to maintain corneal hydration and promote epithelial healing. Topical corticosteroids may be used cautiously to control immune-mediated damage. Given the viral etiology, antiviral eye drops can be prescribed to prevent further viral reactivation. After managing the inflammation, autologous limbal stem cell transplantation may be considered in our patient. Long-term follow-up is essential to monitor disease progression and prevent complications such as corneal scarring and infection.

Limbal stem cells act as a proliferative barrier between the cornea and conjunctiva. Limbal stem cell deficiency (LSCD) is characterized by neovascularization and conjunctivalization of the corneal surface [1].

LSCD can be congenital or develop following physical trauma, chemical burns, thermal injury, surgery, autoimmune disease or infection, particularly viral [2]. Several cases of LSCD have been reported following herpetic keratitis (HSV) [3]. Liu X et al. have reported 4 cases of limbic insufficiency complicating ophthalmic herpes zoster (VZV) [4].

To our knowledge, this is the first case of LSCD complicating chickenpox in a child reported in the literature. This case highlights the importance of recognizing LSCD in pediatric patients with concomitant viral infections, such as chickenpox. Early diagnosis and a collaborative approach between pediatricians and ophthalmologists are essential for effective management.

## Consent for publication

Informed written consent was obtained from the patient’s guardian for publication of this report.
